# Male size, not female preferences influence female reproductive success in a poeciliid fish (*Poecilia latipinna*): a combined behavioural/genetic approach

**DOI:** 10.1186/s13104-018-3487-2

**Published:** 2018-06-08

**Authors:** Ulrike Scherer, Ralph Tiedemann, Ingo Schlupp

**Affiliations:** 1 Unit of Evolutionary Biology/Systematic Zoology, University of Potsdam, Karl-Liebknecht-Str. 24-25, 14476 Potsdam, Germany; 2 Present Address: Institute of Zoology, Universität Hamburg, Martin-Luther-King-Platz 3, 20146 Hamburg, Germany; 3 Department of Zoology, University of Oklahoma, 730 Van Vleet Oval, Norman, OK 73019 USA

**Keywords:** Fitness, Life history, Mate choice, Microsatellite analysis, Offspring weight, Paternity analysis, Sailfin molly, Sexual selection

## Abstract

**Objective:**

We investigated the potential role of indirect benefits for female mate preferences in a highly promiscuous species of live-bearing fishes, the sailfin molly *Poecilia latipinna* using an integrative approach that combines methods from animal behavior, life-history evolution, and genetics. Males of this species solely contribute sperm for reproduction, and consequently females do not receive any direct benefits. Despite this, females typically show clear mate preferences. It has been suggested that females can increase their reproductive success through indirect benefits from choosing males of higher quality.

**Results:**

Although preferences for large body size have been recorded as an honest signal for genetic quality, this particular study resulted in female preference being unaffected by male body size. Nonetheless, larger males did sire more offspring, but with no effect on offspring quality. This study presents a methodical innovation by combining preference testing with life history measurements—such as the determination of the dry weight of fish embryos—and paternity analyses on single fish embryos.

**Electronic supplementary material:**

The online version of this article (10.1186/s13104-018-3487-2) contains supplementary material, which is available to authorized users.

## Introduction

Female mate choice is one of the key drivers of sexual selection [1, 2]. In species where males provide direct benefits to females, this is easily understood. However, in many species such direct benefits are not apparent, yet female choice exists despite clear costs, such as, increased risk of predation, loss of time, and energetic costs [3–8]. To balance the costs of choice, indirect (genetic) benefits have been invoked. They are much harder to document, but are thought to enhance female fitness through an increased reproductive value of the offspring [1, 2, 6, 9]. Despite the immense theoretical support and conceptual relevance of the evolution of female choice for indirect benefits, it still remains controversial how important those effects are (meta-analysis by [10]).

Here, we studied the evolution of female choice for indirect benefits by investigating female mating preference of the sailfin molly, *Poecilia latipinna*. The sailfin molly is a particularly well-suited model species to test female choice for indirect benefits because it is a sexually reproducing and promiscuous species. Males only contribute ejaculates (mainly sperm) to reproduction without providing any direct benefits. Yet, females typically show clear mate preferences, e.g. for large male body size [11, 12]. In the present study, we assessed female preference for two males of different size, which we used as a proxy for male quality. Body size is heritable [13–17], and larger offspring generally have higher fitness in many different taxa [18–22], including fishes [23–25]. After determining female preferences, in our experiment, females were allowed to mate with the two males, a large and a small one. We measured both offspring quantity (number of embryos) and a proxy of offspring quality (embryo dry weight). We investigated three hypotheses: (I) we expected females to prefer larger, higher quality males, (II) we expected larger and preferred males to sire more offspring, (III) and finally, we expected larger and preferred males to sire offspring of higher quality. Furthermore, we wanted to test if it would be possible to utilize ethanol preserved fish embryos for both DNA paternity testing using microsatellites and assessment of life history data (i.e., dry weight after gentle low temperature desiccation). The usage of microsatellite markers is a widely accepted and reliable method to assess parentage (reviewed in [26]). Embryo body weight is an important life history measure, as larger offspring generally have a higher fitness (see above).

## Main text

### Methods

#### Fish collection and maintenance

Fish were collected in August 2013 in Brownsville, Texas (Rio Grande Valley, Cameron County; 25°54.58′N 97°26.61′W), *P. latipinna* (approx. 200–300 individuals; including males and females) were caught using a standard 1.2 m seine, with 0.3 cm mesh width, and transported to the University of Oklahoma in Norman, Oklahoma. Males (*N *= 60) were held in a common tank (60 L). Gravid females (*N *= 42) were isolated and kept in individual tanks (7 L). All fish were maintained under standardised conditions (24–28 °C water temperature, aerated and filtered water, weekly water changes, specific conductance (SPC) of the water: 1300–1800 μS/cm, 12:12 h light:dark cycle). Twice daily the fish were fed ad libitum with flake food in the mornings and frozen mosquito larvae in the evenings.

#### Female mate preference

A detailed description of the procedure for female preference assessment can be found in Additional file 1. Females (*N *= 27) were tested for their mate preference using binary choice tests (standard procedure for testing preferences; [27–29]), as soon as possible after giving birth to a brood (time lag 5–36 h). After parturition, females can be readily fertilised by males for approximately 3–4 days [30, 31]. It was assumed that female choice would be most acute during this phase. Females were allowed to choose between one small (mean ± SE = 29.5 ± 0.3 mm) and one large male (mean ± SE = 34.1 ± 0.6 mm; size difference within male pairs: mean ± SD = 5.0 ± 0.1 mm). Each male pair was only used once. We quantified female preference as the time spent with a male relative to the time spent with both males (please see Additional file 1). The two males presented to a female were defined as the preferred (preference > 50%; mean ± SE = 65.17 ± 10.58%) and the non-preferred male (preference < 50%; mean ± SE = 34.83 ± 10.58%). All males (mean ± SE = 31.8 ± 0.5 cm) and females (mean ± SE = 45.1 ± 0.6 cm) were measured for standard length after mate choice trials.

#### Mating

Immediately after the choice test, females were mated with their preferred and non-preferred male. Mating order was randomized. One of the two males was placed in the female’s tank for 24 h; followed by another 24 h with the other male. Mating experiments were performed in small 7 L tanks to increase the likelihood of a mating through a higher interaction rate. Half of the females were mated with the preferred male first (*N *= 13; with 8 large and 5 small males) and half were mated with the non-preferred male first (*N *= 14, 7 large and 7 small males). This was repeated, so that the overall mating period amounted to 4 days, spanning the entirety of the female’s fertile phase. Because not all females preferred the large male, we were able to cross male size and mating order in this experiment. After the mating period, a small dorsal fin-clip of each male was collected and preserved in 100% ethanol to yield paternal DNA. Twenty-two days after the mating ended, females were euthanized using an overdose of Tricaine methanesulfonate (MS-222) and preserved in 100% ethanol until dissection. Females were euthanized before they could release their brood. A period of 22 days was chosen because gestation of poeciliid fishes takes approximately 30 days [30, 31].

#### Life history measures and paternity analysis

Prior to dissection, a small fin-clip was taken of each female to provide maternal DNA. Females were dissected following the protocol of Riesch et al. [32]. The developmental stage of each embryo was classified following the protocol of Riesch et al. [33]. During dissection and classification, embryos were kept in 100% ethanol and stored separately to prevent degradation and contamination of DNA. Embryos were dried in an incubator oven for 10 days at 40 °C [33] and then placed in a desiccator for 3–4 h to remove residual dampness before embryo dry weight (mg) was measured.

If available, a subset of 20 embryos per female was randomly chosen as representatives for the whole brood (*N *= 110, 15–20 embryos per female). For the paternity analysis, embryos and potential parents were genotyped at 10 unlinked polymorphic microsatellite loci [34], please see Additional file 2.

#### Data analysis

Statistical analyses were performed using R version 3.2 [35]. We tested for a difference in female preference (arcsine-square root-transformed) of small versus large males using a paired t-test (*N *= 27). A t-test power analysis was computed using the R package *pwr* [36]. Further, we fitted a linear regression model (LM) on female preference for large males (*N *= 27; arcsine-square root-transformed) using male size and the size difference between the small and large male within a pair as predictors. We analysed offspring quantity by fitting an LM on the number of offspring sired by each male (*N *= 6). The number of offspring sired by a male equaled a female’s fecundity (i.e., we did not detect multiple paternity). The full model incorporated female preference and size for the father as predictor variables. Offspring quality was analysed by fitting a linear mixed-effect model implemented in the *lme4*-package [37] on the embryo dry weight (*N *= 110 embryos originating from 6 females), including female preference and size of the father as the fixed effects and mother ID as a random effect. For all models, non-significant predictors were eliminated in a stepwise backward procedure. Before analyses predictors were z-transformed (except for the developmental stage) to control for potential non-linear relationships between variables. Prior to generating z-scores, predictors were transformed: number of offspring: square root-transformation; female preference: arcsine-square root-transformation; embryo dry weight: x^2^-transformation; male and female size: log_10_-transformation; [38]. Using preparatory linear regression analyses, we removed the effect of female size on offspring number and the effect of the developmental stage and female size on the embryo dry weight before the analyses. Our data did not show deviations from a normal distribution (Shapiro–Wilk-tests).

### Results

**Fig. 1:**
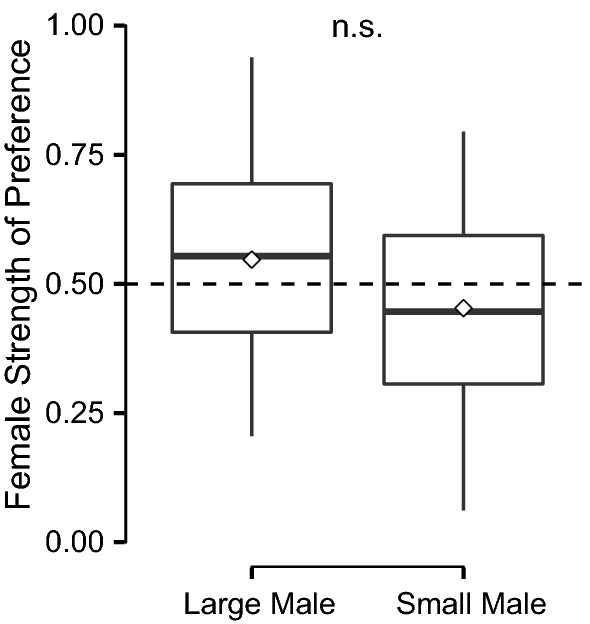
Female preference for male standard length did not deviate from random choice (50%; dashed line). Box plots with 1.5 interquartile range, mean (white diamond), and median (−); *ns* non-significant

We found no significant difference in female preference for small (mean ± SE = 45.29 ± 3.48%) vs. large males (mean ± SE = 54.70 ± 3.48%), (paired t-test; *t*_26_ = 1.3691, *P *= 0.1827, power = 0.7316; Fig. 1). Female preference did neither correlate with male size (linear regression: *F*_1, 25_ = 0.0256, *P *= 0.8742) nor with the size difference between males (linear regression: *F*_1, 25_ = 0.9348, *P *= 0.3429), for female preference data please see Additional file 3.

**Fig. 2:**
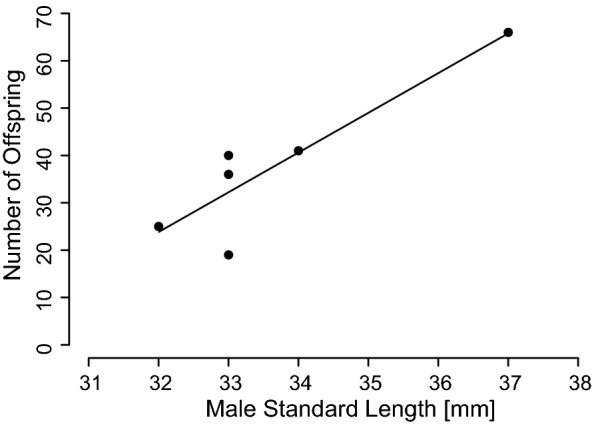
Positive relation between male size and number of offspring sired. Original data presented (*N *= 6)

The number of offspring sired by a male was significantly influenced by male size (LM; *F*_1,4_ = 8.0365, *P *= 0.0471, adjusted R^2^ = 0.5846; Fig. 2). However, this result is based on a low sample size of only six females with offspring (see Additional file 3). Female preference for the father (mean ± SE = 55.48 ± 11.18%), (LM; *F*_1,3_ = 0.5421, *P *= 0.5149) did not affect the number of offspring. All offspring were exclusively sired by the larger male, independent of the female’s behavioural preference (3 females preferred the large male, 3 females preferred the small male), data are given in Additional file 4. Furthermore, there was a strong effect of the mating order on offspring production. Only first males that were large sired offspring. Offspring dry weight was not influenced by male size (LMM; $\chi^{2}_{1}$ = 0.2311, *P *= 0.6307). Also, female preference did not influence the dry weight (LMM; $\chi^{2}_{1}$ = 0.0002, *P *= 0.9901).

### Discussion

Female mating preferences for large male body size are widespread within a broad range of taxa (review by: [39]), with *P. latipinna* being no exception [11, 12, 28, 40, 41], but such a preference was not detected in the present study. Female preferences are based on the recognition of multiple cues, e.g. colouration [42] courtship behaviour [43] or MHC similarity [44–46], which provides a much more powerful assessment of male quality when combined than size does alone. In this study, females received additional visual, behavioural and olfactory cues during mate choice trials, which might have interfered with female preference for large male body size.

Only a subset of females had offspring and due to the low sample size we are interpreting our findings with great caution. Interestingly, however, although male size was not important in determining female preferences, it had an effect on the number of offspring sired by a male. Offspring were sired only by large males and offspring quantity increased with male size, providing evidence that male intrinsic quality may increase female reproductive success. A potential mechanism would be a positive correlation of male size with quality or quantity of sperm. Male size has been shown to correlate with the amount of sperm in the eastern mosquitofish, *Gambusia holbrooki* [47], mandarinfish, *Synchiropus splendidus* [48] and *P. latipinna* [49]. Furthermore, mating order had an effect on male reproductive success with only first males siring offspring indicating first-male precedence. Effects of insemination order on fertilization success are widely distributed in animals with internal fertilization [50, 51]. As the effect of male size and mating order were confounded, it is unclear whether first male precedence or male size per se generated this pattern.

Although offspring quantity was influenced by male size, offspring quality (measured as embryo dry weight) was not. Offspring quality is determined by various components, and to obtain substantial insight into the influence of mate preferences and mate quality on reproductive success, future studies need to consider various aspects of offspring quality, including offspring performance.

### Limitations

Clearly, our study is limited by the low number of successful matings, resulting in a small final sample size. Also, fish were collected at a single collection site not allowing to control for population effects.

## Additional files

**Additional file 1.** Binary choice test. Detailed description for the determination of female preference.

**Additional file 2.** Paternity analysis. Detailed description for the determination of paternity.

**Additional file 3.** Data on female mate choice, mating and fecundity.

**Additional file 4.** Results paternity analysis and offspring life history data.
