# Supplementary material for: Male size, not female preferences influence female reproductive success in a poeciliid fish (Poecilia latipinna): a combined behavioural/genetic approach
Source: BMC Res Notes. 2018 Jun 8;11:364. doi: 10.1186/s13104-018-3487-2 (PMC5994011; doi:10.1186/s13104-018-3487-2)
Supplement: Supplementary file 1 — Additional file 1. Binary choice test. Detailed description for the determination of female preference. [file 13104_2018_3487_MOESM1_ESM.docx]

BINARY CHOICE TEST

To begin a choice test, we introduced one focal female and two males, one small and one large male, into the choice chamber (30 x 60 cm, 30 L). The choice chamber was divided into three equal zones (20 x 30 cm) with a neutral zone in the middle and two preference zones at the adjacent sides. One male was placed in the middle of each preference zone using a clear Plexiglas cylinder with perforations on each side (cylinder size = 8.5 x 8.5 cm, diameter of the holes = 0.3 cm, 28 holes on each side with a distance of 1cm) to allow for chemical communication. The focal female was placed in the middle of the neutral zone using another clear Plexiglas cylinder. After an acclimation time of 10min, the cylinder of the focal female was removed and the test period of 10min started as soon as the female began to move around. The association time (the time a female spent in the preference zone of a male; in sec) was recorded for each male. The trial was repeated after switching sides for the presented males, to control for a potential side-bias. A female was considered side-biased when she spent more than 80% of the time available for preference zones in a single preference zone, regardless of which male was placed there [1, 2]. Female preference was quantified as the total association time (sum of both trials) of a particular stimulus male divided by the total association time with both stimulus males.

REFERENCES

1. Poschadel JR, Plath M, Schlupp I: Divergent female mating preference in a clonal fish. *Acta Ethologica* 2009; 12:55-60.

2. Schlüter A, Parzefall J, Schlupp I: Female preference for symmetrical vertical bars in male sailfin mollies. *Animal Behaviour* 1998; 56:147-153.
