# Supplementary material for: Male size, not female preferences influence female reproductive success in a poeciliid fish (Poecilia latipinna): a combined behavioural/genetic approach
Source: BMC Res Notes. 2018 Jun 8;11:364. doi: 10.1186/s13104-018-3487-2 (PMC5994011; doi:10.1186/s13104-018-3487-2)
Supplement: Supplementary file 2 — Additional file 2. Paternity analysis. Detailed description for the determination of paternity. [file 13104_2018_3487_MOESM2_ESM.docx]

PATERNITY ANALYSIS

Genomic DNA was isolated from parental fin clips (6 mothers and 12 potential fathers) and desiccated embryos using the NucleoSpin Tissue-Kit (Nucleic Acid and Protein Purification, REF 740952.250; produced by Macherey-Nagel). Embryos and potential parents were genotyped at 10 unlinked polymorphic microsatellite loci [GT-I41, GA-III49A, GA-IV42, GT-I34, GA-I29B, GA-V18, GT-II33, GA-I29A, GA-III28, GT-II16, see: 1]. The software Micro-Checker version 2.2.3 [2] was used to control for genotyping errors, such as allele drop outs, or the presence of null alleles.

Paternity was assigned with Cervus version 3.0 [3], using the following parameters: number of candidate fathers = 12, proportion of candidate fathers sampled = 1.000, proportion of loci type d = 1.000, proportion of loci mistyped = 0.010, error rate in likelihood calculations = 0.010, and minimum number of typed loci = 10. A Simulation of the parentage analysis was run on the same set of parameters for a number of 100 000 randomly generated offspring genotypes. Confidence of paternity was assured on a level of 95% using the critical Delta resulting from the simulation analysis.

REFERENCES

1. Tiedemann R, Moll K, Paulus KB, Schlupp I: New microsatellite loci confirm hybrid origin, parthenogenetic inheritance, and mitotic gene conversion in the gynogenetic Amazon molly (*Poecilia formosa*). *Molecular Ecology Notes* 2005; 5:586-589.

2. Van Oosterhout C, Hutchinson WF, Wills DPM, Shipley P: Micro-checker: software for identifying and correcting genotyping errors in microsatellite data. *Molecular Ecology Notes* 2004; 4:535-538.

3. Kalinowski ST, Taper ML, Marshall TC: Revising how the computer program CERVUS accommodates genotyping error increases success in paternity assignment. *Molecular ecology* 2007; 16:1099-1106.
