# Supplementary material for: Male size, not female preferences influence female reproductive success in a poeciliid fish (Poecilia latipinna): a combined behavioural/genetic approach
Source: BMC Res Notes. 2018 Jun 8;11:364. doi: 10.1186/s13104-018-3487-2 (PMC5994011; doi:10.1186/s13104-018-3487-2)
Supplement: Supplementary file 3 — Additional file 3. Data on female mate choice, mating and fecundity. [file 13104_2018_3487_MOESM3_ESM.pdf]

Additional file 3. Data on female mate choice, mating and fecundity.

Female ID	Female Size	Large Male Size	Small Male Size	Time Lage Parturition and Choice Test	Large Male Preference	Small Male Preference	Female Preference	Mating Order	Fecundity
F01	49	37	31	34.50	0.9383	0.0617	Large Male	Large Male First	66
F02	48	44	32	30.00	0.4080	0.5920	Small Male	Small Male First	0
F03	33	30	29	8.50	0.6814	0.3186	Large Male	Small Male First	0
F05	45	34	28	30.50	0.6152	0.3848	Large Male	Small Male First	0
F06	48	37	30	33.00	0.3041	0.6959	Small Male	Small Male First	0
F07	48	34	28	27.50	0.3707	0.6293	Small Male	Large Male First	41
F08	43	36	29	27.50	0.3776	0.6224	Small Male	Large Male First	0
F09	41	38	30	29.00	0.6043	0.3957	Large Male	Large Male First	0
F10	47	37	31	34.50	0.6000	0.4000	Large Male	Small Male First	0
F11	43	34	30	26.00	0.4944	0.5056	Small Male	Small Male First	0
F12	47	35	27	27.50	0.3114	0.6886	Small Male	Large Male First	0
F13	47	33	27	29.00	0.7146	0.2854	Large Male	Large Male First	40
F14	48	36	30	32.00	0.4874	0.5126	Small Male	Large Male First	0
F15	46	32	28	33.00	0.4924	0.5076	Small Male	Large Male First	0
F16	45	35	29	29.00	0.2403	0.7597	Small Male	Small Male First	0
F17	45	34	29	31.00	0.6923	0.3077	Large Male	Small Male First	0
F18	46	33	27	33.50	0.7265	0.2735	Large Male	Large Male First	0
F19	45	34	29	35.00	0.7935	0.2065	Large Male	Small Male First	0
F20	48	32	29	28.00	0.6950	0.3050	Large Male	Large Male First	25
F21	43	33	30	35.00	0.2053	0.7947	Small Male	Large Male First	19
F22	43	32	31	4.00	0.7297	0.2703	Large Male	Large Male First	0
F23	46	30	30	29.50	0.5538	0.4462	Large Male	Small Male First	0
F24	44	30	30	21.00	0.4913	0.5087	Small Male	Small Male First	0
F25	43	32	31	32.00	0.7339	0.2661	Large Male	Small Male First	0
F26	44	32	31	5.00	0.5323	0.4677	Large Male	Large Male First	0
F27	44	32	31	7.50	0.5714	0.4286	Large Male	Large Male First	0
F72	47	33	30	34.00	0.4047	0.5953	Small Male	Large Male First	36
