# Supplementary material for: Male size, not female preferences influence female reproductive success in a poeciliid fish (Poecilia latipinna): a combined behavioural/genetic approach
Source: BMC Res Notes. 2018 Jun 8;11:364. doi: 10.1186/s13104-018-3487-2 (PMC5994011; doi:10.1186/s13104-018-3487-2)
Supplement: Supplementary file 4 — Additional file 4. Results paternity analysis and offspring life history data. [file 13104_2018_3487_MOESM4_ESM.pdf]

Additional File 4. Results paternity analysis and offspring life history data. Life history parameters (dry weigth, developmental stage (DS)) and paternity analysis of a randomly chosen subset of embryos (*N*= 110). IDs denote mother (F) and embryo (E) number. Number of mismatching loci, LOD score and Delta are given for both candidate fathers (small and large male, respectively). The LOD score is the natural logarithm of the likelihood ratio (likelihood that a candidate parent is the true parent divided by the likelihood that this candidate is not the true parent). Delta (difference in LOD scores between the most likely parent and the second most likely parent) was obtained to assign parentage for a critical Delta on a level of 95% = 0.00.

Embryo ID	Embryo Dry Weigth [mg]	Embryo DS	Large Male			Small Male		
			Mismatching Loci	LOD score	Delta	Mismatching Loci	LOD score	Delta
F01-E07	2.1	30	0	3.12	3.12	1	-3.28	0.00
F01-E10	1.6	25	0	1.26	1.26	2	-7.83	0.00
F01-E14	1.9	35	0	1.03	1.03	2	-7.31	0.00
F01-E15	2.1	35	0	1.24	1.24	1	-3.53	0.00
F01-E17	2.1	35	0	0.94	0.94	2	-7.02	0.00
F01-E23	1.8	30	0	1.52	1.52	2	-8.37	0.00
F01-E26	2.2	25	0	0.52	0.52	1	-3.11	0.00
F01-E28	1.8	30	0	3.15	3.15	3	-13.20	0.00
F01-E29	2.7	35	0	4.89	4.89	1	-5.48	0.00
F01-E30	2.5	35	0	1.96	1.96	2	-9.28	0.00
F01-E32	2.4	35	0	0.33	0.33	2	-8.85	0.00
F01-E33	2	30	0	3.03	3.03	3	-11.60	0.00
F01-E35	2.3	30	0	0.06	0.06	1	-3.53	0.00
F01-E40	2.9	35	0	3.55	3.55	1	-3.54	0.00
F01-E45	2.5	30	0	0.95	0.95	3	-12.40	0.00
F01-E46	2.2	25	0	2.10	2.10	2	-8.30	0.00
F01-E47	2.4	30	0	2.78	2.78	1	-3.25	0.00
F01-E53	2.9	35	0	2.10	2.10	3	-12.80	0.00
F01-E54	2.4	35	0	0.22	0.22	2	-7.02	0.00
F01-E55	1.2	35	0	2.71	2.71	1	-3.07	0.00
F07-E06	2.3	35	0	4.33	4.33	1	-2.85	0.00
F07-E07	2.8	30	0	4.29	4.29	1	-3.77	0.00
F07-E09	2.7	30	0	5.64	5.64	2	-8.31	0.00
F07-E10	2.8	30	0	2.20	2.20	1	-4.29	0.00
F07-E11	2.8	30	0	3.18	1.89	0	1.29	0.00

Additional File 4. continued.

Embryo ID	Embryo Dry Weigth [mg]	Embryo DS	Large Male			Small Male		
			Mismatching Loci	LOD score	Delta	Mismatching Loci	LOD score	Delta
F07-E14	2.7	35	0	4.08	4.08	1	-1.43	0.00
F07-E18	2.6	35	0	2.85	2.85	1	-4.95	0.00
F07-E20	2.4	35	0	6.24	6.24	1	-1.10	0.00
F07-E21	2.6	35	0	4.33	4.33	1	-2.85	0.00
F07-E23	2.2	30	0	1.77	1.77	0	-0.92	0.00
F07-E24	2.4	35	0	4.56	4.56	1	-3.33	0.00
F07-E26	2.2	30	0	2.48	2.48	1	-4.01	0.00
F07-E29	2.4	30	0	3.29	3.29	1	-2.93	0.00
F07-E30	2.4	30	0	3.16	3.16	1	-2.95	0.00
F07-E32	2.4	30	0	4.25	4.25	2	-4.96	0.00
F07-E34	2.8	30	0	4.56	4.56	1	-0.95	0.00
F07-E36	2.5	30	0	3.51	3.04	0	0.47	0.00
F07-E37	2.8	35	0	3.06	3.06	1	-3.77	0.00
F07-E39	2.7	30	0	3.80	3.80	1	-2.39	0.00
F07-E40	2.8	30	0	4.22	4.22	1	-1.88	0.00
F13-E09	3	25	0	3.83	3.83	2	-4.13	0.00
F13-E10	2.7	20	0	0.10	0.10	1	-1.92	0.00
F13-E14	2.8	20	0	0.78	0.78	1	-4.19	0.00
F13-E24	2.6	25	0	3.66	0.35	0	3.31	0.00
F13-E25	2.5	20	0	1.26	1.26	1	-6.11	0.00
F13-E26	2.8	20	0	4.04	1.60	0	2.44	0.00
F13-E27	2	25	0	4.35	2.44	0	1.91	0.00
F13-E28	3.1	20	0	0.58	0.58	1	-3.84	0.00
F13-E30	2.7	25	0	3.58	3.58	1	-2.48	0.00
F13-E32	2.9	25	0	0.96	0.96	1	-6.41	0.00
F13-E33	2.9	25	0	3.42	1.20	0	2.21	0.00
F13-E34	2.9	20	0	1.40	1.40	0	-0.25	0.00
F13-E35	2.9	25	0	3.31	2.45	0	0.86	0.00

Additional File 4. continued.

Embryo ID	Embryo Dry Weigth [mg]	Embryo DS	Large Male			Small Male		
			Mismatching Loci	LOD score	Delta	Mismatching Loci	LOD score	Delta
F13-E36	1.4	20	0	-0.40	0.00	1	-3.58	0.00
F13-E37	2.4	25	0	0.05	0.05	0	-1.60	0.00
F13-E38	2.8	25	0	2.17	1.50	0	0.68	0.00
F13-E39	1.8	25	0	5.34	5.34	1	-2.34	0.00
F13-E40	2.9	25	0	1.40	1.40	0	-1.49	0.00
F20-E01	2.7	30	0	0.99	0.99	1	-5.43	0.00
F20-E02	2.9	35	0	1.99	1.99	1	-4.68	0.00
F20-E04	2.3	35	0	2.79	2.79	1	-3.95	0.00
F20-E06	2.2	30	0	1.31	1.31	1	-3.57	0.00
F20-E07	2.6	35	0	1.99	1.99	1	-5.00	0.00
F20-E08	2.9	35	0	1.31	1.31	0	-0.82	0.00
F20-E09	2.6	30	0	1.74	1.74	1	-5.30	0.00
F20-E10	2.6	30	0	3.15	3.15	2	-9.00	0.00
F20-E11	2.3	30	0	5.28	5.28	2	-9.24	0.00
F20-E12	2.5	35	0	3.80	3.80	2	-8.11	0.00
F20-E14	2.6	35	0	5.29	5.29	1	-5.08	0.00
F20-E18	2.9	30	0	4.16	4.16	2	-9.54	0.00
F20-E19	2.7	35	0	4.72	4.72	3	-12.00	0.00
F20-E20	2.9	35	0	3.49	3.49	1	-4.79	0.00
F20-E21	2.7	30	0	3.38	3.38	0	-1.52	0.00
F20-E22	2.7	30	0	1.32	1.32	1	-5.72	0.00
F20-E23	2.2	35	0	4.25	4.25	3	-12.40	0.00
F21-E01	3.2	30	0	3.47	2.21	0	1.26	0.00
F21-E02	3.3	25	0	4.42	4.42	0	-0.78	0.00
F21-E03	2.7	25	0	4.72	3.46	0	1.26	0.00
F21-E04	3.1	30	0	3.49	3.49	0	-0.37	0.00
F21-E05	3.3	25	0	3.57	3.36	0	0.21	0.00
F21-E06	3.2	25	0	2.31	1.85	0	0.46	0.00

Additional File 4. continued.

Embryo ID	Embryo Dry Weigth [mg]	Embryo DS	Large Male			Small Male		
			Mismatching Loci	LOD score	Delta	Mismatching Loci	LOD score	Delta
F21-E07	3.1	25	0	4.56	4.24	0	0.32	0.00
F21-E08	3.2	25	0	4.16	4.16	0	-0.92	0.00
F21-E09	3	30	0	3.88	1.53	0	2.35	0.00
F21-E10	2.7	30	0	5.58	5.58	0	-1.45	0.00
F21-E11	3.1	30	0	3.56	1.63	0	1.93	0.00
F21-E12	3.1	25	0	4.42	4.42	0	-0.78	0.00
F21-E13	2.8	25	0	4.72	3.46	0	1.26	0.00
F21-E14	3	25	0	3.88	1.53	0	2.35	0.00
F21-E15	3.3	25	0	2.50	2.50	0	-0.48	0.00
F72-E04	3.4	35	0	1.00	1.00	1	-2.22	0.00
F72-E08	3.2	35	0	5.28	5.28	2	-7.99	0.00
F72-E11	2.6	35	0	4.28	4.28	1	-2.22	0.00
F72-E12	3.2	30	0	1.00	1.00	1	-2.22	0.00
F72-E13	3	35	1	0.42	0.42	1	-1.86	0.00
F72-E14	3.5	35	0	2.39	2.39	1	-2.77	0.00
F72-E15	3.3	35	0	6.39	6.39	2	-8.67	0.00
F72-E16	3	35	0	4.74	4.74	1	-2.21	0.00
F72-E17	3.3	35	0	4.06	4.06	1	-2.89	0.00
F72-E19	3.2	35	0	4.34	4.34	2	-6.08	0.00
F72-E20	3.3	35	0	3.71	3.71	1	-4.13	0.00
F72-E21	2.8	30	0	8.62	8.62	2	-8.67	0.00
F72-E24	2.9	35	0	5.42	5.42	1	-2.77	0.00
F72-E25	3.3	35	0	4.59	4.59	2	-6.63	0.00
F72-E26	2.5	35	0	6.96	6.96	2	-7.32	0.00
F72-E28	2.5	35	0	2.86	2.86	2	-6.07	0.00
F72-E29	3	30	0	7.87	7.87	3	-12.00	0.00
F72-E31	3.3	35	0	4.12	4.12	3	-11.30	0.00
F72-E32	2.5	35	0	6.69	6.69	2	-6.76	0.00
F72-E35	1.1	35	0	6.55	6.55	2	-7.44	0.00
